# High‐Density‐Cluster Ru Catalyst Substitute for Pt/C Benchmark Improves Membrane Water Electrolysis

**DOI:** 10.1002/advs.78247

**Published:** 2026-10-11

**Authors:** Zhen Yuan, Jia Xu, Zhongchen Gao, Linyan A, Shiying Guo, Jingguo Hu, Zhengshan Tian, Xiaoyong Xu

**Affiliations:** ^1^ College of Physical Science and Technology & Institute of Technology for Carbon Neutralization Yangzhou University Yangzhou China; ^2^ Mathematics and Science College Shanghai Normal University Shanghai China; ^3^ College of Chemistry and Chemical Engineering Pingdingshan University Pingdingshan Henan China

**Keywords:** high current density, hydrogen evolution reaction, pH‐universality, proton exchange membrane water electrolyzer, ruthenium cluster

## Abstract

The hydrogen evolution reaction (HER) is of great importance for the renewable energy conversion to hydrogen fuel via water electrolysis. However, the current benchmark Pt/C catalyst suffers from high cost, pH sensitivity, and deficient durability, severely restricting the large‐scale development of membrane water electrolysis. Herein, we develop a cost‐effective Pt‐free catalyst featuring high‐density, ultrafine Ru nanoclusters modified with CoP and uniformly dispersed on nitrogen‐doped carbon films (NCF). This catalyst achieves not only superior HER activity across a wide pH range but also robust stability at ampere‐level large current densities, manifesting as a competitive alternative to commercial Pt/C. Typically, Ru‐CoP/NCF exhibits an overpotential of only 22 mV at 10 mA cm^−2^ in acidic media, with the mass activity and price activity 1.21 and 2.18 times higher than Pt/C, respectively. A proton exchange membrane water electrolyzer using Ru‐CoP/NCF catalyst delivers a high current density of 1.0 A cm^−2^ at a voltage of merely 1.67 V and sustains long‐term operation for 780 h with a minimal decay rate of 84 µV h^−1^. This Ru‐CoP/NCF catalyst thus demonstrates practical potential for the development of non‐Pt membrane electrodes.

## Graphical abstract

Yuan et al. report a high‐density, monodispersed Ru cluster catalyst featuring interfacial covalent bonds that modulate the electronic structure and optimize intermediate adsorption–desorption, thereby conferring pH‐universal HER activity with ampere‐level current densities and enabling the development of Pt‐free membrane electrodes.

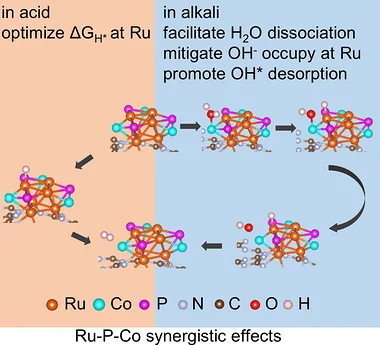

## 1 Introduction

Hydrogen promises an ideal carbon‐free energy carrier to replace fossil fuels toward carbon neutrality [1]. Water electrolysis powered by renewable electricity offers a sustainable route to produce high‐purity hydrogen [2, 3]. Its efficiency and economics mainly rely on the catalytic reaction kinetics on two electrode catalysts. The cathode hydrogen‐evolving reaction (HER), crucial to water splitting [4, 5, 6, 7], generally follows either the Volmer–Heyrovsky or Volmer–Tafel pathways to regulate the hydrogen adsorption and desorption process [8, 9]. The Sabatier principle indicates that an ideal catalyst should bind hydrogen at its active sites neither too weakly nor too strongly, rendering a moderate hydrogen adsorption free energy (ΔG_H*_) [10, 11]. Platinum (Pt) is regarded as the benchmark HER catalyst owing to its near‐optimal hydrogen binding energy [12]. However, its practical deployment is constrained by inherent scarcity and high cost. This limitation is compounded in alkaline media, where the HER kinetics on Pt is severely hampered by the requisite water dissociation step and the subsequent sluggish proton supply [13, 14, 15]. Therefore, developing cost‐effective alternative catalysts that combine robust performance with broad pH applicability is imperative for scalable hydrogen production.

Ruthenium (Ru), a platinum‐group metal costing approximately one‐third the price of Pt [16], has garnered significant interest as a promising Pt alternative for the HER, due to its economic viability and corrosion resistance [17, 18, 19, 20]. However, the practical application of Ru‐based catalysts is hindered by their inadequate activity and stability, particularly under industrial‐level large current densities. On one hand, Ru exhibits an excessive ΔG_H*_ with respect to the Sabatier optimum, which hinders hydrogen desorption and results in suboptimal intrinsic HER activity [21, 22]. In this regard, great efforts have been devoted to modulating the electronic structure of Ru sites to lower ΔG_H*_ through some notable strategies, including heteroatom doping [23, 24], bimetallic alloying [25], and hybridization with transition metal compounds [26, 27, 28], etc. On the other hand, reducing catalytic centers to smaller sizes, such as nanoparticles, clusters, or even single atoms, serves as an effective strategy to enhance the noble‐metal atom utilization [29, 30]. However, the accompanying increase in surface energy as size decreases poses new challenges to the stability of ultrafine catalytic units. To date, few Ru‐based catalysts have been reported that can sustain long‐term operation at ampere‐level current densities in industrial membrane electrode assemblies.

The use of suitable supports to anchor fine Ru units, thereby creating strong metal‐support interactions (MSI) via interfacial bonding and electronic modulation, represents a viable strategy for the concurrent optimization of intrinsic activity and robustness [31, 32, 33]. Recently, 2D porous nitrogen‐doped carbon films (NCF), featuring a large specific surface area, high conductivity, and hydrophilicity, have emerged as excellent candidates for loading metal catalytic units [34, 35]. A central challenge, however, lies in precisely tailoring the coordination environment of metal sites to induce the favorable MSI on NCF [36]. Moreover, achieving a high‐density, monodisperse spatial distribution while minimizing metal unit size is also crucial to go beyond intrinsic activity. However, during the self‐assembly of metal units onto a substrate, it is particularly challenging to simultaneously achieve a configuration that is both small in dimension and high in density.

In this work, we develop a heterogeneous catalyst (Ru‐CoP/NCF) characterized by high‐density dispersion of ultrafine Ru clusters, which are modified with cobalt phosphide (CoP) and uniformly anchored on nitrogen‐doped carbon films (NCF). Benefiting from the structural confinement and electronic modulation imparted by interfacial Ru─P─Co bonds, the Ru‐CoP/NCF catalyst exhibits outstanding HER activity and stability across the full pH range, even at industrial ampere‐level current densities. Moreover, when adopted in a practical proton exchange membrane water electrolyzer (PEMWE), this catalyst requires only 1.67 V cell voltage to drive water splitting at 1.0 A cm^−2^ for over 780 h with negligible attenuation. The results demonstrate both the practicality of our Ru‐CoP/NCF catalyst and its cost‐effectiveness as a substitute for the commercial Pt/C benchmark.

## 2 Results and Discussion

The two‐step synthetic process with melamine and metal ion (Ru^3+^/Co^2+^) sources is facile and scalable, as schematically shown in Figure 1a. First, the Ru^3+^/Co^2+–^melamine mixed precursor was thermally carbonized, yielding an intermediate of highly dispersed bimetallic Ru‐Co clusters on the 2D NCF (denoted as RuCo/NCF). In this step, Ru^3+^/Co^2+^ ions preferentially adsorb to melamine's nitrogen atoms via electrostatic interaction, and then undergo carbothermal reduction to form highly dispersed metal clusters through the templating effect of carbon nitride crystals (Figure S1). Second, the subsequent thermal phosphidation was conducted to drive the modification of Ru clusters with CoP for optimizing interface interactions. Owing to the lower diffusion activation energy, Co atoms readily coordinate with P to form CoP, whereas Ru remains highly dispersed as clusters, resulting in CoP‐decorated Ru clusters with strong MSI. X‐ray diffraction (XRD) patterns and Raman spectra (Figure S2) disclose the conversion of melamine in the mixed precursor into amorphous carbon embedded with tiny Ru crystals.

**FIGURE 1:**
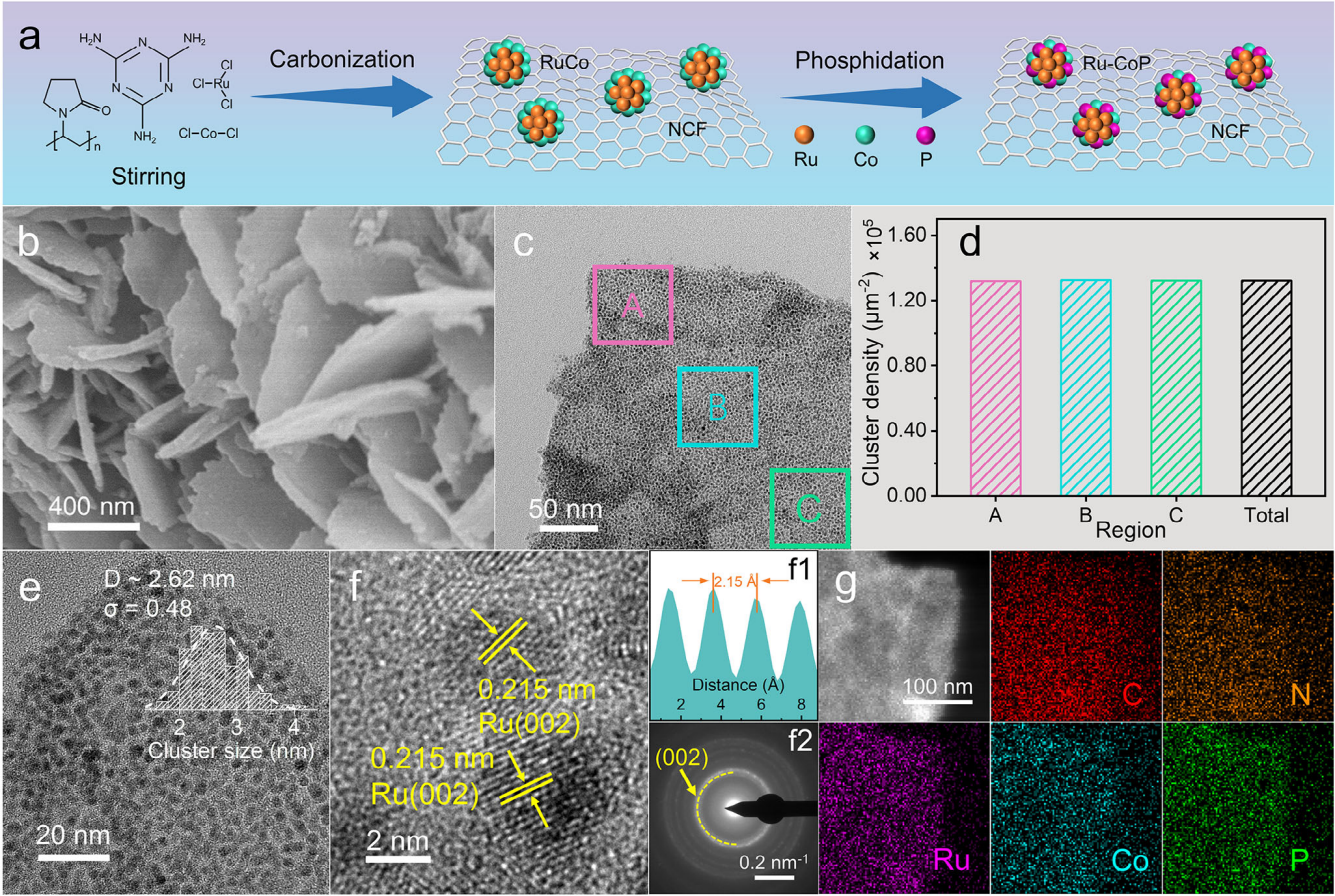
The synthetic procedure and structural characterizations for the Ru‐CoP/NCF composite. (a) Schematic illustration of the synthetic process. (b) SEM image and (c) TEM image. (d) Spatial density of clusters on NCF in the three regions selected by the wireframes in (c). (e) Amplified TEM image with cluster size distribution (inset). (f) HRTEM image showing the lattice fringes, with (f1) the atomic spacing analysis and (f2) the SAED pattern. (g) EDS elemental maps.

Scanning electron microscopy imaging (Figure 1b) shows that the resultant Ru‐CoP/NCF composite exhibits a 2D sheet‐like morphology. Transmission electron microscopy (TEM) analysis (Figure 1c) further reveals that these sheets are adorned with a multitude of ultrafine nanoclusters. Particle density analysis across three distinct regions in Figure 1c (Figure S3) reveals a uniform particle distribution, achieving a balance between good dispersion and high density. The average particle density is calculated to be 1.32 × 10^5^ µm^−2^ (Figure 1d), which is much higher than that 7.05 × 10^4^ µm^−2^ of the commercial 20% Pt/C catalyst (Figure S4), thanks to the smaller particle size and more regular distribution in Ru‐CoP/NCF. The amplified TEM image (Figure 1e) shows uniform particles with a narrow size range of 1–4 nm centered at 2.62 nm (inset). High‐resolution TEM (HRTEM) image (Figure 1f) shows that the nanoclusters are confined within the amorphous matrix. The measured lattice fringes of 2.15 Å within these clusters (Figure 1f1) can be indexed to the (002) plane of metallic Ru, consistent with the selected‐area electron diffraction (SAED) pattern (Figure 1f2). The inverse fast Fourier transform (IFFT) image (Figure S5) reveals the regular atomic arrangement within the cluster and the distorted atomic arrangement at the boundaries, implying interfacial conjugation effects. Energy‐dispersive spectroscopy (EDS, Figure S6) and elemental mapping (Figure 1f) indicate the uniform distribution of Ru, Co, P, C, and N elements in the final Ru‐CoP/NCF composite. The loading of metal Ru was measured to be 36.57 wt.% using inductively coupled plasma mass spectrometry (ICP‐MS, Table S1).

In addition, control samples including Ru/NCF, RuP/NCF, Co/NC, and CoP/NC were synthesized from single metal sources using the same procedure. When only Co ions were present, the carbonized melamine exhibited a nanofiber morphology (Figure S7). In contrast, only when Ru ions were introduced did melamine evolve into 2D nanosheets decorated with Ru clusters (Figure S8). These results demonstrate that the introduction of Ru and the subsequent formation of clusters are critical for directing the carbonization of melamine into 2D NCF, ultimately yielding the unique architecture of cluster‐dispersed 2D nanosheets.

X‐ray photoelectron spectroscopy (XPS) was taken to probe the coordination and electronic states of Ru‐CoP/NCF. The survey spectra verify the existence of C, N, O, Ru, Co, and P elements (Figure S9). As shown in the high‐resolution C 1s+Ru 3d XPS spectra in Figure 2a, during the carbonization process, the characteristic C─N═C coordination of melamine completely transforms into C─O/C─N and C─C coordination, indicating the formation of carbon nitride, accompanied by the full reduction of adsorbed Ru^3+^ ions (282.0 and 286.0 eV) to Ru^0^ species (280.7 and 284.1 eV) [37]. After the phosphidation treatment, the Ru 3d peaks slightly shift toward higher binding energies, suggesting that the electron densities of interfacial Ru atoms decrease via Ru─P bonding [38]. Furthermore, the Ru 3p XPS spectra (Figure 2b) also reveal the reduction of Ru^3+^ (463.3 and 485.4 eV) to Ru^0^ (461.3 and 483.5 eV), along with the subsequent emergence of trace Ru─P coordination (462.6 and 484.9 eV) [19]. In the Co 2p XPS spectra (Figure S10), precursor Co^2+^ ions are partially reduced to Co^0^ and are subsequently transformed into Co─O (781.9 and 797.7 eV) and Co─P (778.6 and 793.6 eV) species after phosphidation [22]. The P 2p and N 1s XPS spectra (Figure S11) reveal P─Co/P─Ru bonds (129.7 and 130.7 eV), P─O species (133.5 and 134.5 eV), as well as pyridinic N (399.0 eV), pyrrolic N (399.8 eV), and graphitic N (401.4 eV) components in Ru‐CoP/NCF.

**FIGURE 2:**
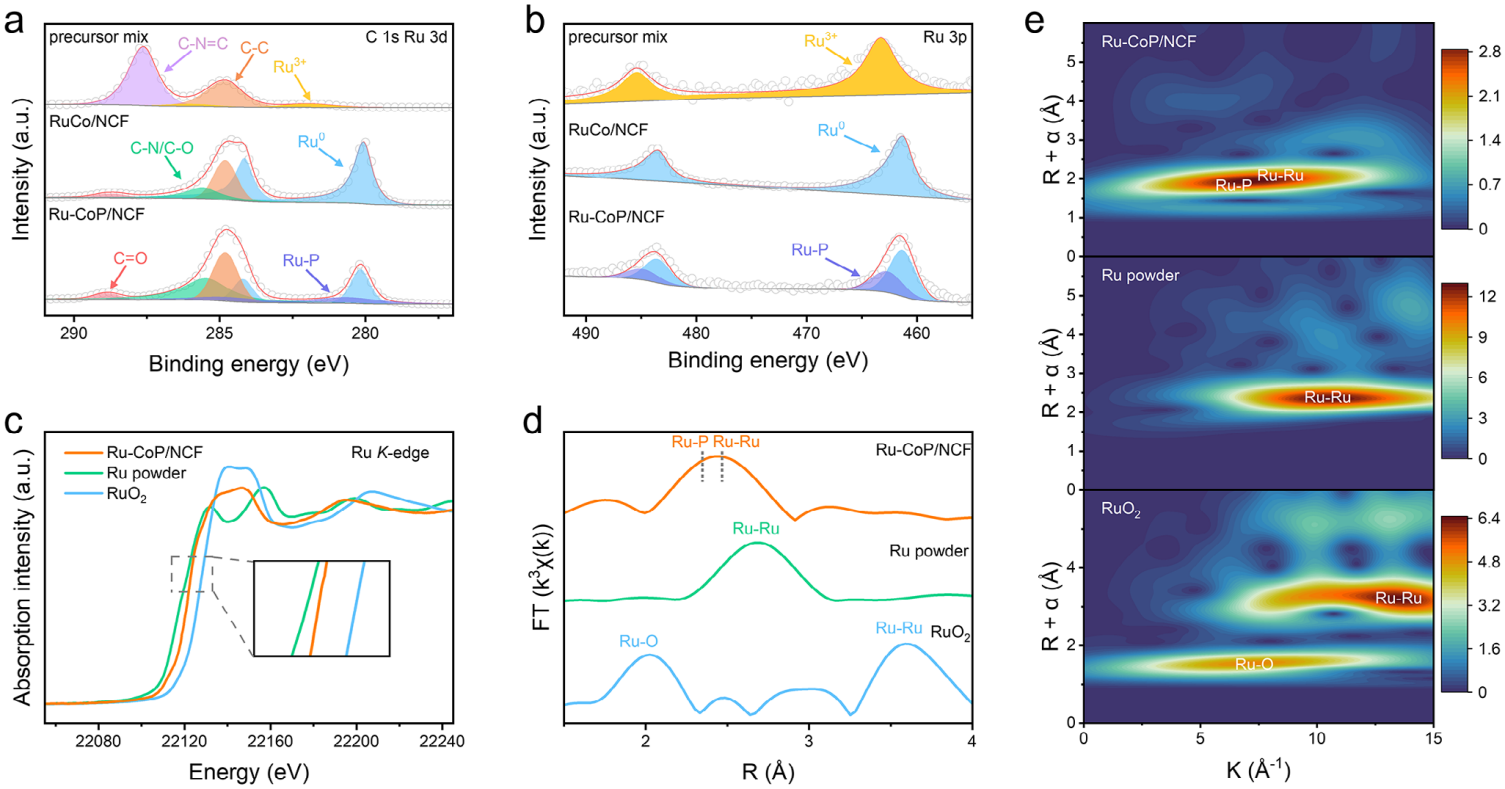
The coordination structure analysis for the Ru‐CoP/NCF composite. High‐resolution XPS spectra at (a) C 1s + Ru 3d and (b) Ru 3p regions for the precursor mixture, intermediate RuCo/NCF, and Ru‐CoP/NCF. (c) XANES spectra at the Ru K‐edge, (d) FT‐EXAFS spectra, and (e) WT‐EXAFS spectra at the Ru K‐edge for Ru‐CoP/NCF, Ru powder, and RuO_2_.

X‐ray absorption near‐edge structure (XANES) and extended X‐ray absorption fine structure (EXAFS) at the Ru K‐edge were investigated for the Ru‐CoP/NCF composite, with metal Ru and RuO_2_ as two references. In the normalized XANES spectra (Figure 2c), the Ru K‐edge absorption energy of Ru‐CoP/NCF lies between those of Ru and RuO_2_, suggesting an oxidation state of Ru between 0 and +4. Quantitative analysis (Figure S12) further determines the average valence to be approximately +1.19. The Fourier‐transformed (FT) k^3^‐weighted‐EXAFS spectrum of Ru‐CoP/NCF (Figure 2d) reveals a characteristic Ru─Ru coordination peak at around 2.45 Å [39], which is distinct from those of RuO_2_ and metallic Ru, indicating a strong confinement effect of Ru clusters within the substrate. An additional Ru─P coordination peak is observed at approximately 2.34 Å at the side [40]. Quantitative curve‐fitting analysis (Figure S13) yields coordination numbers (CN) of 8.47 and 2.65 for the Ru─Ru and Ru─P shells, respectively (Table S2). Furthermore, the wavelet transform (WT) of Ru K‐edge EXAFS oscillations for Ru‐CoP/NCF (Figure 2e) exhibits a prominent intensity feature centered at 7.05 Å^−1^, corresponding to the Ru─Ru scattering shell, which is clearly distinguishable from those observed for RuO_2_ and metallic Ru. Collectively, these structural analyses confirm that small Ru clusters are chemically anchored within the amorphous NCF matrix through Ru─P─Co bonding and strong MSI, which are expected to enhance the electrochemical HER performance.

The electrocatalytic HER performance of the Ru‐CoP/NCF catalyst was assessed using a standard three‐electrode system in three different electrolytes, including 0.5 M H_2_SO_4_ (acid, pH = 0), 1 M PBS (neutral, pH = 7), and 1 M KOH (alkali, pH = 14), respectively. As shown in Figure 3a, the linear sweep voltammetry (LSV) polarization curves indicate that Ru‐CoP/NCF exhibits better HER activity compared to Ru/NCF and Pt/C catalysts, requiring overpotentials of only 22, 8, and 15 mV to achieve a geometric current density of 10 mA cm^−2^ in acidic, alkaline, and neutral media, respectively (Figure 3b). Correspondingly, Tafel slopes of the Ru‐CoP/NCF catalyst are also the smallest, with values of 13.66, 18.04, and 43.18 mV dec^−1^ in acidic, alkaline, and neutral media, respectively (Figure 3c). Tafel slopes below 30 mV dec^−1^ in acidic and alkaline conditions suggest the favorable Volmer‐Tafel mechanism (Figure 3d) [41]. The markedly increased Tafel slope in neutral media originates from slowed kinetics due to poor ionic conduction. Notably, when shifting from acid to alkali, the Pt/C catalyst suffers a significant increase in both overpotential and Tafel slope, due to sluggish proton supply limited by water dissociation kinetics [15]. In contrast, Ru‐CoP/NCF shows minimal sensitivity to electrolyte pH, maintaining its Tafel‐step limiting kinetics even in alkaline media. This pH‐universal activity is crucial for stability under high‐current‐density operation, where local pH fluctuations are inevitable. Based on its superior Tafel slope and overpotential metrics, the Ru‐CoP/NCF catalyst ranks among the first‐rate Ru‐based catalysts reported to date, whether in acidic (Figure 3e and Table S3), alkaline (Figure S14 and Table S4), and neutral media (Figure S15 and Table S5). Remarkably, compared with the commercial 20% Pt/C catalyst, Ru‐CoP/NCF exhibits significant enhancements of both mass activity (Figure 3f) and price activity (Figure 3g), underscoring its high cost‐effectiveness. Moreover, this advantage is particularly pronounced in an alkaline environment, where the performance of Pt/C catalyst is far constrained by limited proton supply. Consequently, the pH‐universal Ru‐CoP/NCF catalyst is suitable not only for proton‐exchange membrane (PEM) water electrolyzers but also for anion‐exchange membrane (AEM) systems.

**FIGURE 3:**
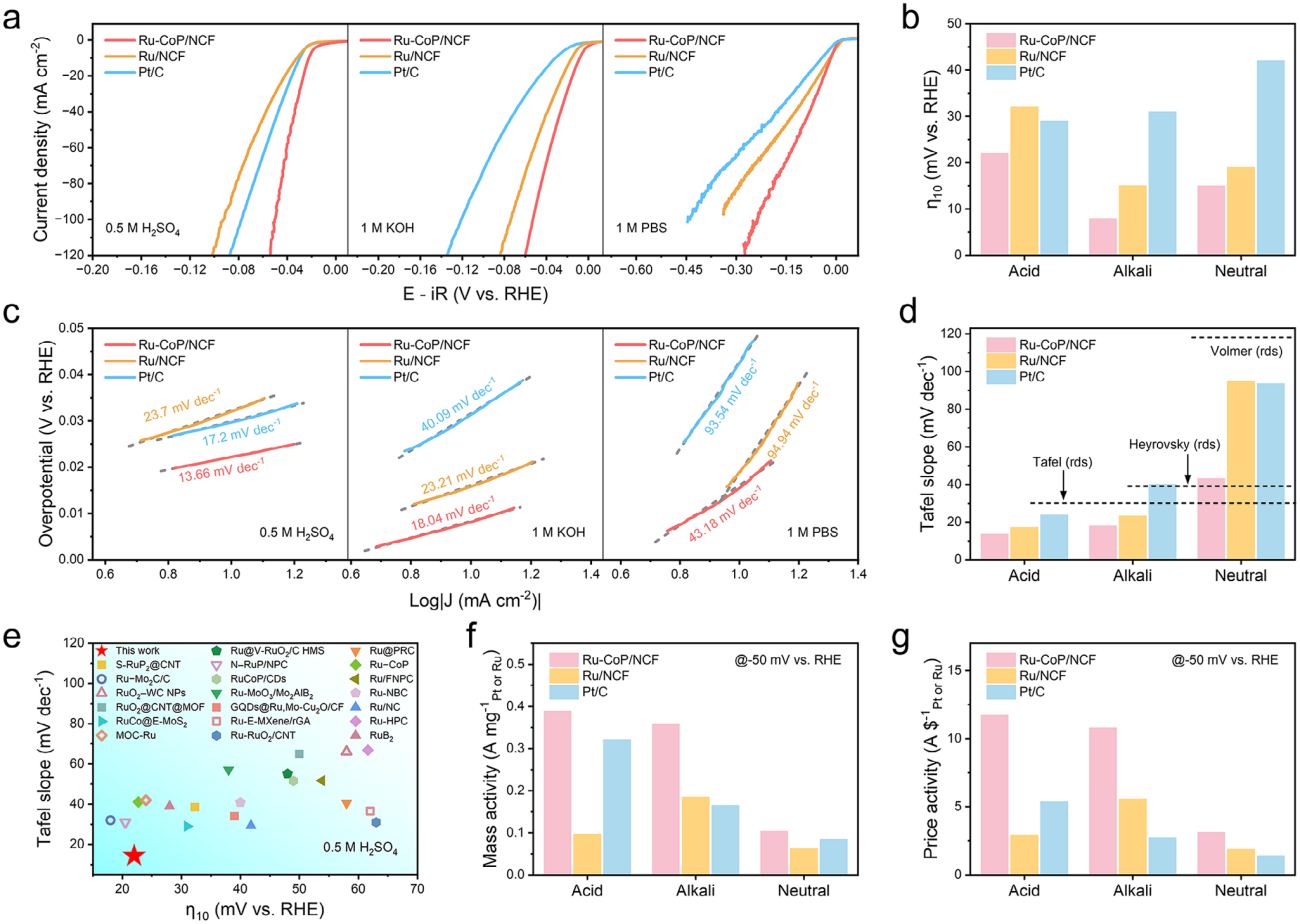
The pH‐universal HER performance. (a) LSV curves of Ru‐CoP/NCF, Ru/NCF, and commercial Pt/C catalysts measured in 0.5 M H_2_SO_4_, 1 M KOH, and 1 M PBS, respectively, and (b) the comparison of overpotentials (η_10_) at 10 mA cm^−2^. (c) Tafel plots of Ru‐CoP/NCF, Ru/NCF, and Pt/C catalysts in three different electrolytes, and (d) the comparison of Tafel slopes. (e) Comparison of overpotential and Tafel slope of Ru‐CoP/NCF with other Ru‐based catalysts reported for the HER in acid. Comparison of (f) mass activity and (g) price activity of Ru‐CoP/NCF with Ru/NCF and Pt/C.

The initial LSV curves without iR compensation and the electrochemical impedance spectra (EIS) are presented in Figure S16 (Table S6). Among all samples compared, Ru‐CoP/NCF demonstrates the most favorable polarization behavior and the smallest interfacial charge‐transfer resistance. Interestingly, Co/NC and CoP/NCF, which lack Ru, show negligible polarization, confirming that Ru clusters serve as the essential active sites for the HER. Double‐layer capacitance (C_dl_) and electrochemical active surface area (ECSA) were derived from non‐Faradaic cyclic voltammetry (CV) (Figure S17), in which Ru‐CoP/NCF exhibits higher C_dl_ (84.5 mF cm^−2^) and ECSA (265.5 cm^2^) compared to Ru/NCF and Pt/C. At an overpotential of −50 mV, the ECSA‐normalized current density (j_ECSA_) and turnover frequency (TOF) of Ru‐CoP/NCF reach 0.047 mA cm^−2^ and 11.9 s^−1^ (Figure S18), respectively, which are higher than those of Ru/NCF (0.023 mA cm^−2^; 5.6 s^−1^) and Pt/C (0.023 mA cm^−2^; 5.7 s^−1^). Furthermore, the exchange current density (j_0_) of Ru‐CoP/NCF (0.26 mA cm^−2^) also exceeds those of Ru/NCF (0.19 mA cm^−2^) and Pt/C (0.12 mA cm^−2^) (Figure S19), further underscoring its exceptional intrinsic HER activity.

Beyond activity, stability is another critical metric for HER catalysts. The Ru‐CoP/NCF catalyst was evaluated via chronopotentiometry (CP) in 0.5 M H_2_SO_4_. As shown in Figure S20, it maintains stable operation at 10 mA cm^−2^ for over 450 h. In situ ICP‐MS reveals minimal metal ion leaching, particularly with an extremely low Ru dissolution rate of 0.001 µg h^−1^. Post‐test structural analysis (Figure S21) further confirms the catalyst's robustness, showing a well‐preserved morphology, unchanged elemental composition, and consistent size and crystal phase of the Ru clusters.

To identify the active centers, thiocyanate ions (SCN^−^) were employed as a metal site‐specific poisoning agent for LSV measurements in acidic and alkaline electrolytes (Figure 4a,b and Figure S22 and Table S7) [42]. The significant activity loss for both Ru‐CoP/NCF and Ru/NCF upon SCN^−^ addition confirms Ru sites as primary HER active centers. Further, the poisoning effect was quantified by the deactivation rate (R_d_), defined as the overpotential increase at 10 mA cm^−2^ (Figure 4c). Notably, Ru/NCF exhibits a markedly lower R_d_ in alkali than in acid, since OH^−^ adsorbed on Ru sites hinders SCN^−^ poisoning. In contrast, Ru‐CoP/NCF retains a pronounced R_d_ in alkali, indicating that the adjacent CoP component mitigates strong OH^−^ adsorption on Ru and thereby maintains the accessibility of Ru sites [43, 44]. Consequently, the enhanced kinetics and pH‐universal activity of Ru‐CoP/NCF originate from the Ru‐CoP synergy. Complementing this insight, CV studies in acid further probe hydrogen adsorption–desorption behavior (Figure 4d). Ru‐CoP/NCF exhibits an earlier onset of underpotential‐deposited hydrogen (upd‐H) adsorption and a larger hydrogen adsorption capacity compared to Ru/NCF. This profile facilitates rapid H* formation at lower overpotentials while preventing excessively strong binding, thus optimizing H* coverage and desorption [45]. Operando CV curves show that the hydrogen desorption potential (des‐H) peaks shift to higher potentials with the increase of scan rates (Figure S23). The hydrogen desorption kinetics can be quantified by plotting the des‐H peak position vs. scan rate and comparing the fitted slopes [46]. The Ru‐CoP/NCF exhibits a lower slope value of 1.11 × 10^−4^ than that of Ru/NCF (2.77 × 10^−4^), which indicates accelerated hydrogen desorption kinetics on Ru‐CoP/NCF. Operando CV curves in alkali also show that Ru‐CoP/NCF remains a more favorable hydrogen adsorption and desorption kinetics compared to Ru/NCF (Figure S24). In addition, in situ EIS was conducted to investigate the charge‐transfer kinetics [47]. The lower phase angles in the low‐frequency region in the Bode plots for Ru‐CoP/NCF compared to Ru/NCF (Figure 4e and Figure S25), and their more rapid decline with increasing potentials, signify faster interfacial charge transfer. This further corroborates the enhanced HER kinetics by the Ru‐CoP synergy.

**FIGURE 4:**
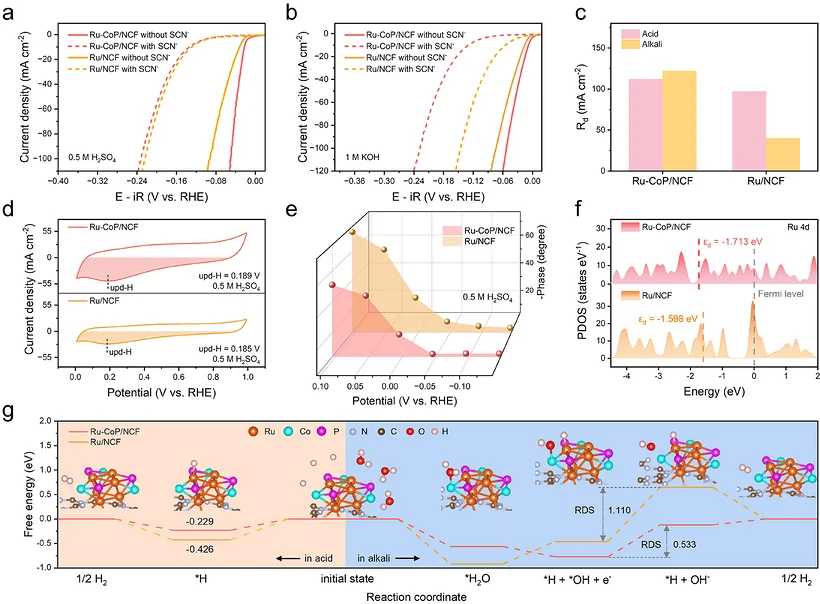
Mechanical insights into enhanced HER performance. LSV curves of Ru‐CoP/NCF and Ru/NCF measured in (a) 0.5 M H_2_SO_4_ and (b) 1 M KOH with/without SCN^−^ agent. (c) Comparison of R_d_ of Ru‐CoP/NCF and Ru/NCF in acid and alkali. (d) CV curves of Ru‐CoP/NCF and Ru/NCF in acid. (e) In situ EIS Bode phase angle as a function of external potentials of Ru‐CoP/NCF and Ru/NCF in acid. (f) PDOS of Ru‐4d orbitals for Ru‐CoP/NCF and Ru/NCF, with the labels of ε_d_ and Fermi level locations. (g) Free energy diagrams of the HER pathways on Ru‐CoP/NCF and Ru/NCF in acid (toward the left) and alkali (toward the right).

Furthermore, density functional theory (DFT) calculations were conducted to uncover the relationship between the electronic structure and intrinsic activity of Ru sites. The theoretical models of Ru‐CoP/NCF and Ru/NCF were constructed and stabilized through relaxation (Figure S26). The different charge density maps in Figure S27 visualize the decreased electron density around Ru sites in Ru‐CoP/NCF compared to Ru/NCF. The projected density of states (PDOS) reveals that Ru‐CoP/NCF has a lower d‐band center (ε_d_) of −1.713 eV compared to −1.598 eV for Ru/NCF (Figure 4f), attributed to the electronic modulation of Ru by adjacent CoP, which is consistent with the XPS analysis. The decline of ε_d_ is further verified by the experimental valence band spectroscopy (VBS) (Figure S28), despite a discrepancy between the theoretical and experimental values [30]. Figure 4g shows free energy diagrams for HER pathways in acid (toward the left) and alkali (toward the right) on Ru‐CoP/NCF and Ru/NCF. The acidic HER involves the hydrogen adsorption and desorption steps, where Ru‐CoP/NCF exhibits a near‐zero ΔG_H*_ value (−0.229 eV) compared to Ru/NCF (−0.426 eV) and reaches an optimized balance between hydrogen adsorption and desorption. The favorable ΔG_H*_ of Ru‐CoP/NCF is attributed to its lower ε_d_, which increases the occupation of antibonding orbitals and effectively reduces the hydrogen adsorption strength. The alkaline HER involves a multi‐step process from water molecule adsorption and dissociation to hydrogen desorption (Figure S29). The spillover of the OH* intermediate serves as the rate‐determining step (RDS) for alkaline HER on both Ru‐CoP/NCF and Ru/NCF. The corresponding free‐energy barrier for Ru‐CoP/NCF is only 0.533 eV, substantially lower than that of Ru/NCF (1.110 eV). Moreover, the water‐dissociation step changes from an endothermic uphill process on Ru/NCF to an exothermic spontaneous process on Ru‐CoP/NCF. This favorable transition arises because CoP preferentially adsorbs water molecules and facilitates their dissociation into H* on Ru sites and OH* on Co sites. Such synergistic cooperation not only facilitates water dissociation, thereby ensuring a sufficient proton supply for the alkaline HER, but also mitigates the adsorption of OH^−^ on Ru sites in alkaline media while concurrently promoting OH* desorption. Collectively, these findings demonstrate that the CoP synergy is advantageous for both H* adsorption–desorption and water‐dissociation/OH*‐desorption processes, ultimately endowing the catalyst with outstanding pH‐universal HER performance.

We prepared a membrane electrode assembly (MEA) using Ru‐CoP/NCF as cathode catalyst (∼0.50 mg cm^−2^) and IrO_2_ as anode catalyst (∼1.25 mg cm^−2^) to examine the practical viability of Ru‐CoP/NCF catalyst in a flow‐type PEMWE (Figure 5a,b). Another PEMWE was also assembled using commercial benchmarks of 40% Pt/C (∼0.50 mg cm^−2^) and IrO_2_ (∼1.25 mg cm^−2^) as cathode and anode catalysts. The Ru‐CoP/NCF ‖ IrO_2_ electrolyzer needs cell voltages of only 1.67 and 1.78 V to achieve industrially‐relevant current densities of 1.0 and 2.0 A cm^−2^ (Figure 5c), which are lower than those of the commercial Pt/C ‖ IrO_2_ couple. Furthermore, the Ru‐CoP/NCF ‖ IrO_2_ electrolyzer demonstrates stable operation at a constant current of 1.0 A cm^−2^ for over 780 h, with a negligible degradation rate of 84 µV h^−1^ (Figure 5d). Meanwhile, in situ ICP‐MS tracking (Figure S30) reveals minimal Ru dissolution with respect to Ir, indicating that the slight degradation is primarily attributed to the deterioration of IrO_2_ anode. In addition, post‐mortem SEM/EDS images of both catalyst layers in the used MEA show that they remain intact, with no visible catalyst detachment (Figure S31). The measured Faraday efficiency through the fluid‐displacement method (Figure S32) is approximately 98.9% (Figure 5e and Table S8 and Note S1), indicating overall water splitting without any side reactions. This performance makes our Ru‐CoP/NCF ‖ IrO_2_ rank among the state‐of‐the‐art PEMWEs reported in the data (Figure 5f and Table S9). Note that the cost of the Ru‐CoP/NCF cathode was estimated to be approximately 58.06$ g^−1^, which is significantly lower than commercial Pt/C catalysts (Tables S10 and S11). The power consumption of the Ru‐CoP/NCF ‖ IrO_2_ at 1.0 A cm^−2^ was calculated to be 4.04 kW h Nm^−3^ H_2_, with a high energy conversion efficiency of 74.3% (Note S2). Further, the hydrogen production cost was calculated to be $0.9 per gallon of gasoline equivalent (GGE), Which Meets the 2026 Target of $2.0/GGE Set By the U.S. Energy Department [48]. Through comparing the Overall Performance in Figure 5g, the Ru‐CoP/NCF ‖ IrO_2_ electrolyzer far surpasses the Pt/C ‖ IrO_2_ couple, suggesting its superior performance and high cost‐effectiveness for hydrogen production

**FIGURE 5:**
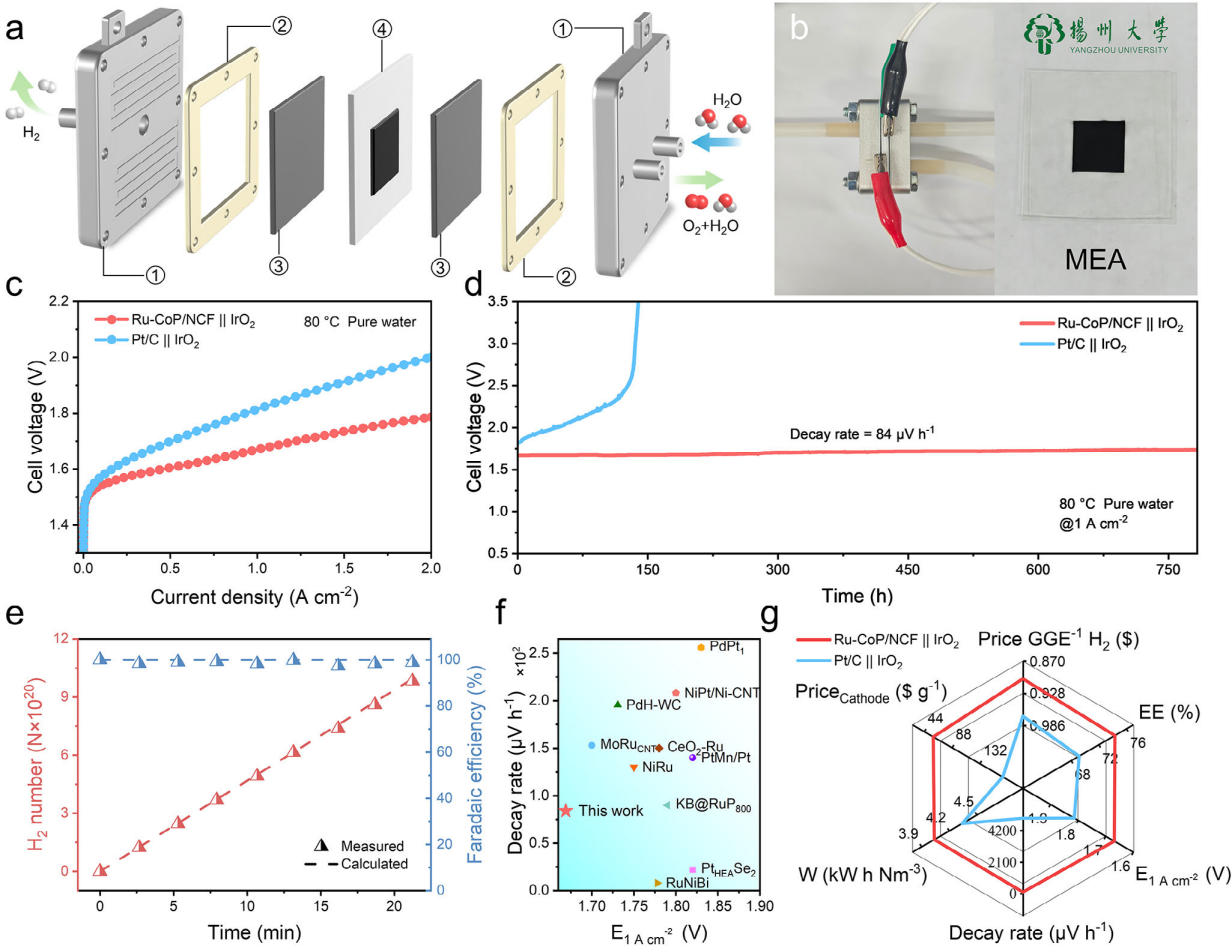
Practical water‐splitting performance over PEMWE. (a) Schematic diagram of PEMWE. Note: ① bipolar plate, ② seal ring, ③ gas diffusion layer, ④ MEA. (b) Photos of the home‐made PEMWE and MEA. (c) Polarization curves of different PEMWEs using Ru‐CoP/NCF and Pt/C cathodes. (d) Long‐term CP curves of two different PEMWEs at 1 A cm^−2^. (e) Faraday efficiency of water splitting over Ru‐CoP/NCF ‖ IrO_2_. (f) Performance comparison of Ru‐CoP/NCF ‖ IrO_2_ with other advanced PEMWEs in terms of cell voltage and decay rate. (g) Comparison of the comprehensive performance of two different PEMWEs.

## 3 Conclusion

In summary, we develop a highly cost‐effective Ru‐CoP/NCF catalyst for the HER, in which ultrafine Ru clusters are highly densely dispersed with 2D NCF and electronically regulated via CoP modification. This catalyst exhibits exceptional pH‐universal HER activity and industrial ampere‐scale stability, along with high mass activity and price activity, pointing it as a competitive alternative to currently commercial Pt/C catalysts. Combined experimental and theoretical analyses reveal that the high‐density dispersion of active units and the optimized adsorption strength of key intermediates together are responsible for the catalyst's superior performance. Moreover, the Ru‐CoP/NC catalyst demonstrates stable long‐term operation in an actual PEMWE device, achieving a hydrogen production cost as low as $0.9/GGE, below the 2026 technical target of $2.0/GGE set by the U.S. Department of Energy.

## Author Contributions

**Zhen Yuan**: investigation, validation, visualization, writing – original draft, data curation. **Jia Xu**: investigation, visualization, writing – review and editing, methodology. **Zhongchen Gao**: software, investigation, visualization, writing – review and editing, formal analysis, methodology. **Linyan A**: investigation, writing – review and editing, visualization. **Shiying Guo**: methodology, software, writing – review and editing, visualization, formal analysis. **Jingguo Hu**: formal analysis, supervision, project administration, writing – review and editing. **Zhengshan Tian**: formal analysis, funding acquisition, software, methodology, validation, writing – review and editing, resources. **Xiaoyong Xu**: conceptualization, writing – original draft, writing – review and editing, funding acquisition, project administration, validation, data curation, supervision, formal analysis.

## Conflicts of Interest

The authors declare no conflicts of interest.

## Supporting information




**Supporting File**: advs78247‐sup‐0001‐SuppMat.docx.

## Data Availability

The data that support the findings of this study are available from the corresponding author upon reasonable request.
