# Supplementary material for: High‐Density‐Cluster Ru Catalyst Substitute for Pt/C Benchmark Improves Membrane Water Electrolysis
Source: Adv Sci (Weinh). 2026 Oct 11:e78247. Online ahead of print. doi: 10.1002/advs.78247 (PMC13655321; doi:10.1002/advs.78247)
Supplement: Supplementary file 1 [file ADVS-9999-e78247-s001.docx]

**Supplementary Information**

**High-Density-Cluster Ru Catalyst Substitute for Pt/C Benchmark Improves** **Membrane Water Electrolysis**

*Zhen Yuan,*^1^ *Jia Xu,*^1^ *Zhongchen Gao,*^1^ *Linyan A, Shiying Guo, Jingguo Hu, Zhengshan Tian,* and Xiaoyong Xu**

Dr. Z. Yuan, Dr. J. Xu, Dr. L. A, Dr. S. Guo, Prof. J. Hu, Prof. X. Xu

College of Physical Science and Technology & Institute of Technology for Carbon Neutralization, Yangzhou University, Yangzhou 225002, China.

E-mail: [xxy@yzu.edu.cn](mailto:xxy@yzu.edu.cn)

Prof. Z. Tian

College of Chemistry and Chemical Engineering, Pingdingshan University, Pingdingshan, Henan, 467000, China.

E-mail: tianzhengshan@163.com

Dr. Z. Gao

Mathematics and Science College, Shanghai Normal University, Shanghai 200233, China

[1] These authors contributed equally to this work.

**Experimental section**

***Materials.*** Ruthenium(III) chloride trihydrate (RuCl₃·3H₂O) and commercial Pt/C (20 wt% and 40 wt%) were obtained from Shanghai Macklin Biochemical Technology Co., Ltd. Nafion perfluorinated resin solution was purchased from Suzhou Sinero Technology Co., Ltd. Hydrogen peroxide (H₂O₂, 30 wt%), sulfuric acid (H₂SO₄, 98 wt%), melamine (C₃H₆N₆), cobalt(II) chloride hexahydrate (CoCl₂·6H₂O), sodium hypophosphite monohydrate (NaH₂PO₂·H₂O), polyvinylpyrrolidone K30 ((C₆H₉NO)ₙ), isopropanol (C₃H₈O), and ethanol (C₂H₆O) were all supplied by Shanghai Hushi Laboratory Equipment Co., Ltd. Deionized water was prepared in-house.

***Preparation of mixture precursor.*** First, 2.4 g of C₃H₆N₆, 100 mg of RuCl₃·3H₂O, 18 mg of (C₆H₉NO)ₙ, and 626 mg of CoCl₂·6H₂O were dissolved in 80 mL of deionized water. The resulting solution was then transferred to a hot plate, heated to 55 °C, and continuously stirred for 30 minutes. Afterward, the mixture was collected by centrifugation and washed repeatedly with deionized water and ethanol. The obtained product was finally dried in a vacuum oven at 60 °C for 12 h.

***Synthesis of Ru-CoP/NCF and control samples.*** The Ru-CoP/NCF catalyst was prepared through a two-step process involving carbonization followed by phosphidation. First, the precursor mixture containing RuCl₃·3H₂O, C₃H₆N₆, CoCl₂·6H₂O, and (C₆H₉NO)ₙ was synthesized according to the procedure described above. For carbonization, 1.4 g of the precursor was placed in an alumina boat and heated in a tube furnace at 600 °C for 1 h under an argon flow of 50 sccm, yielding RuCo/NCF. Subsequently, the as-obtained RuCo/NCF was subjected to phosphidation via chemical vapor deposition (CVD). Typically, 300 mg of NaH₂PO₂ was placed in an alumina boat positioned at the upstream zone of the furnace, while another boat containing 50 mg of RuCo/NCF was placed 7 cm downstream. The furnace was heated to 350 °C and maintained for 1 h under an argon flow of 100 sccm, during which phosphine vapor generated from NaH₂PO₂ was transported onto the sample, affording Ru-CoP/NCF.

For comparison, a series of control samples were synthesized using analogous procedures. Ru/NCF was prepared by carbonizing a precursor mixture without cobalt source (i.e., RuCl₃ + C₃H₆N₆ mixed precursor) under the same carbonization conditions. The resulting Ru/NCF was then phosphidated following the identical CVD method to obtain RuP/NCF. Similarly, Co/NC was obtained by carbonizing a precursor mixture without ruthenium source (i.e., CoCl₂ + C₃H₆N₆ mixed precursor), and subsequent phosphidation of Co/NC under the same conditions yielded CoP/NC.

***Characterization.*** XRD analysis was performed on a Shimadzu X-7000 diffractometer using Cu Kα radiation (λ = 1.54 Å) with a scanning rate of 5° min⁻¹. SEM images were obtained on a Hitachi S-4800II instrument at an acceleration voltage of 5 kV. TEM observations were conducted on a Tecnai F30 microscope operated at 100 kV, equipped with an EDS module for elemental analysis. XPS measurements were carried out on an Escalab 250Xi spectrometer using Al Kα radiation (1486.60 eV). ICP-MS analysis was performed on an Elan DRC-e (PerkinElmer) instrument. XAFS spectroscopy was conducted at the BL14B2 beamline of the SPring-8 synchrotron facility (8 GeV, 100 mA), Japan.

***Fabrication of membrane electrode assemblies.*** A piece of Nafion 115 proton exchange membrane (N115, DuPont) with dimensions of 3 cm × 3 cm was sequentially pretreated by immersion in a 5 wt% hydrogen peroxide solution and then in a 5 wt% sulfuric acid solution, each at 80 °C for 1 h. The membrane was subsequently soaked in deionized water at 80 °C for 1 h and stored in deionized water prior to use. For catalyst preparation, commercial IrO₂ was used as the anode catalyst, while commercial 40% Pt/C and the as-synthesized Ru‑CoP/NCF were employed as cathode catalysts. The anode catalyst ink was prepared by dispersing 2.5 mg of commercial IrO₂ in a mixture of 10 mL deionized water, 15 mL isopropanol, and 184 μL of 5 wt% Nafion solution under ultrasonication until a homogeneous dispersion was obtained. The cathode catalyst ink was prepared following the same procedure, except that 1.0 mg of the target catalyst (either Ru‑CoP/NCF or 40% Pt/C) was dispersed in the mixed solvent with the identical composition. To fabricate the MEAs, the as-prepared anode and cathode inks were separately sprayed onto opposite sides of the pretreated N115 membrane using an ultrasonic spray coater, with a geometric spraying area of 1 cm² on each side. The catalyst-coated membrane was subsequently hot-pressed at 10 MPa for 3 min. The catalyst loading was determined by calculating the spraying loss, which was approximately 50%.

***Electrochemical measurements.*** All electrochemical tests were conducted on a CHI660E electrochemical workstation using a standard three-electrode system. The measurements were performed in 0.5 M H₂SO₄ (pH = 0), 1.0 M KOH (pH = 14), and 1.0 M phosphate-buffered saline (PBS, pH = 7) solutions. For the HER tests, an Ag/AgCl electrode with 3.5 M KCl was employed as the reference electrode in acidic and neutral electrolytes, while a Hg/HgO electrode was used in alkaline medium. A graphite rod served as the counter electrode for all pH conditions. To prepare the working electrode, a catalyst slurry was first formulated by mixing 4.8 mg of the powder sample, 1.2 mg of acetylene black, 300 μL of deionized water, 300 μL of ethanol, and 50 μL of 5 wt% Nafion solution. After thorough ultrasonication to form a homogeneous ink, 12 μL of the dispersion was drop-cast onto a pre-polished glassy carbon electrode (GCE, diameter: 4 mm) and allowed to dry at room temperature. The resulting catalyst-modified GCE was used as the working electrode for all electrochemical measurements. Polarization curves were recorded at a scan rate of 1 mV s⁻¹ within appropriate potential windows. EIS measurements were carried out over a frequency range from 10⁻² to 10⁵ Hz.

All potentials were iR-corrected and converted to the reversible hydrogen electrode (RHE) scale. The iR-corrected potential ($E_{\mathrm{cor}}$) was calculated using the equation $E_{\mathrm{cor}}=E(V vs. RHE)-iR_{s}$, where $E\left( V vs. RHE \right)=E\left( V vs. RE \right)+0.0592\times pH+E^{0}(V vs. RE)$, with $R_{s}$ representing the experimentally measured series resistance and $E^{0}$ denoting the standard potential of the reference electrode relative to the standard hydrogen electrode (SHE). Tafel slope (b) was derived by fitting the linear portion of the Tafel plot to the equation $\eta=blog|j|+a$. The exchange current density ($j_{0}$) was calculated by the equation $\log\left| j_{0} \right|=a/b$.

The $\mathrm{ECSA}$ was determined from the $C_{\mathrm{dl}}$ using the equation $ECSA=C_{\mathrm{dl}}/C_{s}\times S$ (cm²), where $C_{s}$ is the specific capacitance of the electrode (40 μF cm⁻²) and $S$ represents the geometric area of the glassy carbon electrode (0.12566 cm²). CV measurements were performed at various scan rates within the non-Faradaic potential region. The $C_{\mathrm{dl}}$ value was derived by plotting ($j_{a}-j_{c})/2$ against the scan rate at a fixed potential within this region, where $j_{a}$ and $j_{c}$ correspond to the anodic and cathodic current densities, respectively. The $\mathrm{ECSA}$-normalized current density was then calculated as $j_{\mathrm{ECSA}}=i/ECSA$ (mA cm⁻²), with $i$ being the measured current.

The mass activity toward HER, normalized by the noble metal content, was evaluated at an overpotential of −0.05 V vs. RHE. Specifically, the mass activity of Ru or Pt was calculated as $MA=i/m$ (A mg⁻¹), where $i$ is the measured current (A) and $m$ is the mass of Ru or Pt loaded on the electrode. Similarly, the price-normalized activity (PA) was obtained by dividing the measured current by the cost corresponding to the noble metal loading, i.e. $PA=i/P$ (A dollar⁻¹), where $P$ represents the price of the Ru or Pt loaded on the electrode.

The TOF, defined as the number of H₂ molecules evolved per second per active site, was estimated using the following equations: $TOF=(j\times A)/(2\times F\times N)$ (s⁻¹) and $N=(C_{\mathrm{dl}}\times A\times\Delta V)/(2F)$ (mol), where $j$ is the current density (A cm⁻²), $A$ is the electrode area (cm²), the factor 2 accounts for the two electrons involved in generating one H₂ molecule, $F$ is the Faraday constant (96485.3 C mol⁻¹), and $N$ is the number of active sites (mol). $\Delta V$ represents the potential window of the CV scan (V), and the product$(C_{\mathrm{dl}}\times A\times\Delta V)$ corresponds to the voltammetric charge ($Q$, C). The factor 1/2 accounts for the charge–discharge cycle characteristics of CV curves.

***Theoretical calculations.*** DFT calculations were performed using the Vienna Ab Initio Simulation Package (VASP). The electron exchange-correlation interactions were described by the Perdew–Burke–Ernzerhof (PBE) functional within the generalized gradient approximation (GGA). Core electrons were treated using the projector augmented wave (PAW) method, and van der Waals interactions were accounted for by employing the DFT-D3 correction scheme. A plane-wave cutoff energy of 400 eV and a 3 × 3 × 1 Monkhorst–Pack k-point mesh were used for Brillouin zone sampling. Structural relaxations were carried out until the force on each atom was less than 0.01 eV Å⁻¹ and the energy change was below 10⁻⁴ eV. A vacuum layer of 20 Å was introduced along the z-direction to avoid periodic interactions between adjacent slabs.

The Gibbs free energy change ($\Delta G$) for each elementary step was calculated using the equation $\Delta G=\Delta E+{\Delta E}_{\mathrm{ZPE}}-T\Delta S$, where $\Delta E$ is the electronic energy difference obtained from DFT, ${\Delta E}_{\mathrm{ZPE}}$ and $\Delta S$ represent the zero-point energy correction and entropy change, respectively. These quantities were derived from vibrational frequency calculations performed at 298.15 K. The d-band center ($\varepsilon_{d}$) of Ru was computed by integrating the projected density of states (PDOS) according to the equation $\varepsilon_{d}=(\int_{-\infty}^{E_{F}} E\rho_{d}\left( E \right)dE)/(\int_{-\infty}^{E_{F}} \rho_{d}\left( E \right)\mathrm{dE})$, where $\rho_{d}(E)$ denotes the d-orbital projected density of states at energy $E$, and $E_{F}$ is the Fermi level. To elucidate interfacial charge transfer in the stable adsorption configurations, charge density difference ($\Delta\rho$) was analyzed using an equation $\Delta\rho=\rho_{\mathrm{total}}-\rho_{a}-\rho_{b}$, where $\rho_{\mathrm{total}}$ is the total charge density of the adsorption system, while $\rho_{a}$ and $\rho_{b}$ are the charge densities of the isolated substrate and the adsorbed molecule, respectively, each fixed at the same geometry as in the combined system.

**Figure S1.** Scanning electron microscopy (SEM) images of (a) the precursor mixture and (b) intermediate RuCo/NCF after the first-step carbonization. (c-e) Transmission electron microscopy (TEM) images with different scales, and (f) energy dispersive spectroscopy (EDS) elemental maps of RuCo/NCF.

**Figure S2.** (a) XRD patterns and (b) Raman spectra of the mixed precursor, intermediate RuCo/NCF, and resultant Ru-CoP/NCF.

**Figure S3.** (a-c) Statistical analysis of cluster distribution densities in the typical A, B, C regions and (d) whole region in Figure 1c.

**Figure S4.** (a-c) TEM images with different scales of commercial 20% Pt/C catalyst, with a size distribution (inset) in (b). (d) The corresponding statistical analysis of spatial distribution density of Pt particles, with a comparison on particle size and density for Pt/C and Ru-CoP/NCF (inset).

**Figure S5.** HRTEM image and the corresponding IFFT image (inset) of Ru-CoP/NCF.

**Figure S6.** EDS spectrum of Ru-CoP/NCF.

**Figure S7.** SEM images of (a) CoCl_2_+C_3_H_6_N_6_ mixed precursor, (b) Co/NC and (c) CoP/NC.

**Figure S8.** SEM images of (a) RuCl_3_+C_3_H_6_N_6_ mixed precursor, (b) Ru/NCF, and (c) RuP/NCF. (d,e) TEM images, (f) HRTEM image, and (g) EDS maps of Ru/NCF.

**Figure S9.** XPS survey spectrum of Ru-CoP/NCF.

**Figure S10.** High-resolution XPS spectra at Co 2p region for the precursor mixture, RuCo/NCF, and Ru-CoP/NCF.

**Figure S11.** High-resolution XPS spectra at (a) P 2p and (b) N 1s regions of Ru-CoP/NCF.

**Figure S12.** Ru valence state derived from ΔXANES spectra for Ru-CoP/NCF.

**Figure S13.** Ru K-edge EXAFS fitting curves for Ru-CoP/NCF, shown in (a) the k-space and (b) R-space.

**Figure S14.** Comparison on overpotential and Tafel slope of Ru-CoP/NCF with other Ru-based catalysts reported for the HER in alkaline media (Table S4).

**Figure S15.** Comparison on overpotential and Tafel slope of Ru-CoP/NCF with other Ru-based catalysts reported for the HER in neutral media (Table S5).

**Figure S16.** (a-c) Original LSV curves without iR compensation and (d-f) EIS Nyquist plots of Ru-CoP/NCF, Ru/NCF, Pt/C, RuCo/NCF, RuP/NCF, Co/NC, and CoP/NC samples measured in 0.5 M H_2_SO_4_, 1 M KOH and 1 M PBS, respectively.

**Figure S17.** CV curves at various scan rates measured in 0.5 M H_2_SO_4_ for (a) Ru-CoP/NCF, (b) Ru/NCF, and (c) Pt/C catalysts. (d) The estimated $C_{\mathrm{dl}}$ values via fitting of the relationship between current density difference and scan rates, and (e) a comparison of ECSA values for Ru-CoP/NCF, Ru/NCF, and Pt/C catalysts.

**Figure S18.** Normalized (a) $j_{\mathrm{ECSA}}$ and (b) TOF for Ru-CoP/NCF, Ru/NCF, and Pt/C catalysts. Comparison on (c) $j_{\mathrm{ECSA}}$ and (d) TOF values at -50 mV for different catalysts.

**Figure S19.** (a) Intercepts of Tafel slopes and (b) the estimated $j_{0}$ values for for Ru-CoP/NCF, Ru/NCF, and Pt/C catalysts.

**Figure S20.** (a) Stability testing in CP at 10 mA cm^-2^ in 0.5 M H_2_SO_4_ for Ru-CoP/NCF catalyst, and (b) metal-leached amounts measured by in situ ICP-MS.

**Figure S21.** (a-c) TEM and HRTEM images, with (c1) the lattice atom intensity and (c2) SAED pattern for Ru-CoP/NCF catalyst after stability testing. The inset in (b) shows a cluster size distribution. (d) EDS spectrum and (e) elemental maps of Ru-CoP/NCF after stability testing.

**Figure S22.** (a,b) Original LSV curves without iR compensation and (c,d) EIS Nyquist plots of Ru-CoP/NCF and Ru/NCF measured in a) 0.5 M H_2_SO_4_ and b) 1 M KOH with SCN^-^ agent.

**Figure S23.** CV curves at different scan rates of (a) Ru-CoP/NCF and (b) Ru/NCF in 0.5 M H_2_SO_4_. (c) The des-H peak position as a function of CV scan rate.

**Figure S24.** CV curves at different scan rates of (a) Ru-CoP/NCF and (b) Ru /NCF in 1 M KOH. (c) Plots of des-H peak position versus scan rates. (d) CV curves at 50 mV s^-1^ of Ru-CoP/NCF and Ru /NCF for the comparison of des-H peak positions and proton-accessible electrochemical areas (PAEA).

**Figure S25.** Bode phase plots of in situ EIS for (a,b) Ru-CoP/NCF and (c,d) Ru/NCF in 0.5 H_2_SO_4_ and 1 M KOH.

**Figure S26.** Atomic structure models of Ru-CoP/NCF (-H*) and Ru/NCF (-H*) for DFT calculations.

**Figure S27.** Different charge density maps of Ru-CoP/NCF and Ru/NCF. The contours in yellow and blue colors denote charge accumulation and depletion, respectively.

**Figure S28.** VBS spectra of (a) Ru-CoP/NCF and (b) Ru/NCF.

**Figure S29.** Schematic diagrams of alkaline HER pathways on (a) Ru-CoP/NCF and (b) Ru/NCF.

**Figure S30.** Time-dependent Ru and Ir contents in the electrolyte during CP test at 1.0 A cm^-2^ over Ru-CoP/NCF ‖ IrO_2_ electrolyzer.

**Figure S31.** Post-mortem SEM images of (a) cathode and (b) anode sides of the used MEA, with EDS maps of Ru and Ir elements (insets).

**Figure S32.** Photo of the setup for on-line measuring H_2_ and O_2_ yields over Ru-CoP/NCF ‖ IrO_2_ operated at 250 mA cm^-2^ using water displacement method.

**Table S1.** Elemental contents in Ru-CoP/NCF composite by the ICP-MS.

**Table S2.** Local structure parameters around Ru estimated by EXAFS analysis.

CN: coordination numbers; R: bond distance; σ^2^: Debye-Waller factors; ΔE: the inner potential correction; R factor: goodness of fit.

**Table S3.** Performance comparison of the acidic HER for Ru-CoP/NCF with other advanced Ru-based catalysts reported in literature.

**Table S4.** Performance comparison of the alkaline HER for Ru-CoP/NCF with other advanced Ru-based catalysts reported in literature.

**Table S5.** Performance comparison of the neutral HER for Ru-CoP/NCF with other advanced Ru-based catalysts reported in literature.

**Table S6.** $R_{s}$ values of different electrodes in three electrolytes.

**Table S7.** $R_{s}$ values of Ru-CoP/NCF and Ru/NCF in 0.5 M H_2_SO_4_ and 1 M KOH with SCN^-^ agent.

**Table S8.** Numbers of reaction electrons and conduction electrons, as well as the calculated Faraday efficiency (FE) for water electrolysis over Ru-CoP/NCF ‖ IrO_2_.

**Table S9.** Performance comparison of Ru-CoP/NCF ‖ IrO_2_ with reported PEMWEs in literature.

**Table S10.** Material costs for preparing Ru-CoP/NCF catalyst.

**Table S11.** Cost comparison of Ru-CoP/NCF catalyst with commercial Pt/C catalysts.

**Note S1.** Calculation details of Faraday efficiency.

Faradaic efficiency (FE) was determined from the total passed charge and evolved gas volume during continuous electrolysis at 250 mA cm⁻² for over 21 min. H₂ and O_2_ from the PEMWE with Ru-CoP/NCF (cathode) and IrO_2_ (commercial anode) were collected via water displacement method. The H_2_/O_2_ volume ratio stayed close to the theoretical 2:1 throughout the operation process. Quantitative analysis of actual gas production showed good consistency with the theoretical H_2_ and O_2_ yields, rendering a near-unity FE of 98.9% for the Ru-CoP/NCFⅡIrO_2_.

The calculation formula for FE in this experiment is as follows:

$\mathrm{FE}\left( H_{2} \right)=\frac{Reaction electrons}{Conduction electrons}\times100\%=\frac{{(V}_{H_{2}}\div V_{m})\times N_{A}\times2}{t\times I\div e}\times100\%$,

where $V_{H_{2}}$, $N_{A}$, $t$, $I$ and $e$ represent the measured hydrogen volume (L), the Avogadro constant (6.022×10^23^ mol^−1^), the reaction time (s), the reaction current (A) and the elementary charge (1.602×10^−19^ C), respectively. $V_{m}$ denotes the molar volume of gas at standard atmospheric pressure and 25 °C (24.5 L mol^−1^).

**Note S2.** Calculation details for the performance parameters of electrolyzers.

H_2_ production rate@1 A cm^-2^:

$R=\frac{j\times FE}{2\times e\times N_{A}}=\frac{1\times0.989}{2\times1.602\times{10}^{-19}\times6.022{\times10}^{23}}=5.12582{\times10}^{-6}$ mol H_2_ cm^-2^ s^-1^

Lower heating value (LHV) of H_2_:

$$LHV=120 \text{kJ}\text{ g}^{\text{-1}}\text{ }\text{H}_{\text{2}}=2.42\times{10}^{5}\text{ J }\text{mol}^{\text{-1}}\text{ }\text{H}_{\text{2}}$$

H_2_ power density:

$P1=R\times LHV=5.12582{\times10}^{-6}\mathrm{mol}\text{H}\text{2}\text{ cm}\text{-2}\text{ s}\text{-1}\times2.42 \times{10}^{5} \text{J }\text{mol}^{\text{-1}}\text{ }\text{H}_{\text{2}}=1.24045$ W cm^-2^

Power density of Ru-CoP/NCF ‖ IrO_2_:

$P2=j\times V=1 \text{A}\text{ cm}^{\text{-2}}\times1.669 V=1.669$ W cm^-2^

Energy efficiency of Ru-CoP/NCF ‖ IrO_2_:

$\mathrm{EE}=\frac{P1}{P2}=\frac{1.24045 W \mathrm{cm}^{-2}}{1.669 W \mathrm{cm}^{-2}}\times100\%=74.323$ %

H_2_ production price of Ru-CoP/NCF ‖ IrO_2_:

$\text{P }\left( \text{\$/GGE} \right)\text{ H}_{\text{2}}\text{ }=\frac{1 GGE H_{2}}{R} \times P2\times Electricity bill =\frac{0.997\mathrm{kg}}{5.12582{\times10}^{-6} \text{mol }\text{H}_{\text{2}}\text{ }\text{cm}^{\text{-2}}\text{ s}^{\text{-1}}\times2 \text{g }\text{mol}^{\text{-1}}} \times1.669 W \mathrm{cm}^{-2}\times\frac{\$ 0.02}{1 \text{kW h}}=\$ 0.90175$GGE^-1^

Volume rate of H_2_ production@1 A cm^-2^ of Ru-CoP/NCF ‖ IrO_2_:

$R_{V}=\text{R}\times22.4 \text{L }\text{mol}^{\text{-1}}=5.12582{\times10}^{-6}\mathrm{mol}\text{H}\text{2}\text{ cm}\text{-2}\text{ s}\text{-1}\times22.4 \text{L }\text{mol}^{\text{-1}}=1.14818{\times10}^{-4}$ L H_2_ cm^-2^ s^-1^

Energy consumption per Nm^3^ H_2_ of Ru-CoP/NCF ‖ IrO_2_:

$W=\frac{P2}{R_{V}}=\frac{1.669 \text{W }\text{cm}^{\text{-2}}}{1.14818{\times10}^{-4} \text{L} \text{H}\text{2}\text{ cm}\text{-2}\text{ s}\text{-1 }\times3600}=4.04$ KW h Nm^-3^ H_2_
